# Machine Learning–Based Prediction of Growth in Confirmed COVID-19 Infection Cases in 114 Countries Using Metrics of Nonpharmaceutical Interventions and Cultural Dimensions: Model Development and Validation

**DOI:** 10.2196/26628

**Published:** 2021-04-23

**Authors:** Arnold YS Yeung, Francois Roewer-Despres, Laura Rosella, Frank Rudzicz

**Affiliations:** 1 Department of Computer Science University of Toronto Toronto, ON Canada; 2 Vector Institute for Artificial Intelligence Toronto, ON Canada; 3 Dalla Lana School of Public Health University of Toronto Toronto, ON Canada; 4 Unity Health Toronto Toronto, ON Canada

**Keywords:** COVID-19, machine learning, nonpharmaceutical interventions, cultural dimensions, random forest, AdaBoost, forecast, informatics, epidemiology, artificial intelligence

## Abstract

**Background:**

National governments worldwide have implemented nonpharmaceutical interventions to control the COVID-19 pandemic and mitigate its effects.

**Objective:**

The aim of this study was to investigate the prediction of future daily national confirmed COVID-19 infection growth—the percentage change in total cumulative cases—across 14 days for 114 countries using nonpharmaceutical intervention metrics and cultural dimension metrics, which are indicative of specific national sociocultural norms.

**Methods:**

We combined the Oxford COVID-19 Government Response Tracker data set, Hofstede cultural dimensions, and daily reported COVID-19 infection case numbers to train and evaluate five non–time series machine learning models in predicting confirmed infection growth. We used three validation methods—in-distribution, out-of-distribution, and country-based cross-validation—for the evaluation, each of which was applicable to a different use case of the models.

**Results:**

Our results demonstrate high *R*^2^ values between the labels and predictions for the in-distribution method (0.959) and moderate *R*^2^ values for the out-of-distribution and country-based cross-validation methods (0.513 and 0.574, respectively) using random forest and adaptive boosting (AdaBoost) regression. Although these models may be used to predict confirmed infection growth, the differing accuracies obtained from the three tasks suggest a strong influence of the use case.

**Conclusions:**

This work provides new considerations in using machine learning techniques with nonpharmaceutical interventions and cultural dimensions as metrics to predict the national growth of confirmed COVID-19 infections.

## Introduction

### Background

In response to the COVID-19 pandemic, national governments have implemented nonpharmaceutical interventions (NPIs) to control and reduce the spread in their respective countries [1-5]. Indeed, early reports suggested the potential effectiveness of the implementation of NPIs to reduce the transmission of COVID-19 [2,4-8] and other infectious diseases [9-11]. Many epidemiological models that forecast future infection numbers have therefore suggested the role of NPIs in reducing infection rates [2,4,7,12], which can aid the implementation of national strategies and policy decision-making. Recent research incorporates publicly available data with machine learning for use cases such as reported infection case number forecasting [13-16]. Although these studies have used various features, such as existing infection statistics [13], weather [14], media and internet activity [15], and lockdown type [16], to predict infection case numbers, no study has yet examined the combination of NPIs and cultural dimensions in predicting infection growth. In this paper, we include the implementation of NPIs at the national level as features (ie, independent variables) in predicting the national growth of the number of confirmed infection cases. Based on recent studies that identify cultural dimensions as having influence in the effectiveness of NPIs [17-19], we also incorporate cultural dimensions as features. Prior work has focused on NPI variations in different regions of specific countries [2,5,6,20,21]. In contrast, our study involves 114 countries.

Various metrics may provide different perspectives and insights on the pandemic. In this study, we focus on one: confirmed infection growth (CIG), which we define as the 14-day growth in the cumulative number of reported infection cases. Other common metrics to measure the transmission rates of an infectious disease are the basic reproduction number, *R*_0_, which measures the expected number of direct secondary infections generated by a single primary infection when the entire population is susceptible [3,22] and the effective reproduction number, *R_t_* [2], which accounts for immunity within a specified population. Although such metrics are typically used by epidemiologists as measures of the transmission of an infectious disease, these metrics are dependent on estimation model structures and assumptions; therefore, they are application-specific and can potentially be misapplied [22]. Furthermore, the public may be less familiar with such metrics as opposed to more practical and observable metrics, such as the absolute or relative change in cumulative reported cases.

### Related Work

Mathematical modelling of the transmission of infectious disease is a common method to simulate infection trajectories. A common technique for epidemics is the susceptible-infected-recovered (SIR) model, which separates the population into three subpopulations (susceptible, infected, and recovered) and iteratively models the interaction and shift between these subpopulations, which change throughout the epidemic [23,24]. Variations of this model have since been introduced to reflect other dynamics expected of the spread of infectious diseases [25-27]. These variations of the SIR model have also been applied to the ongoing COVID-19 pandemic [28-31].

The recent increase in data availability through advances in the internet and other data sources has enabled the inclusion of other factors in epidemiology modelling [32,33]. Since the early months of the COVID-19 pandemic, Johns Hopkins University has managed the COVID-19 Data Repository by the Center for Systems Science and Engineering (CSSE), which aggregates daily statistics of reported infection and mortality numbers across multiple countries [34]. Data sets related to governmental policies and NPIs have also been released publicly on the web. Notable COVID-19–related data sets include the Oxford COVID-19 Government Response Tracker (OxCGRT) [1], Complexity Science Hub COVID-19 Control Strategies List [35], CoronaNet [36], county-level socioeconomic data for predictive modeling of epidemiological effects (US-specific) [37], and CAN-NPI (Canada-specific) [20]. Additional COVID-19 data sets relate to social media activity [38-41], scientific publications [42-44], population mobility [45-48], and medical images [49-52]. In this work, we focus on the use of NPIs in the forecast of COVID-19 infection growth. Specifically, we selected the CSSE data set for infection statistics and the OxCGRT for NPI features due to their global comprehensiveness. Although features can be extracted from additional COVID-19 data sets in our models, we limited the scope of this study to COVID-19 NPI features.

Recent research has also linked the effect of cultural dimensions in responses to the COVID-19 pandemic. Studies suggest that cultural dimensions may affect individual and collective behavior [53-57] and the effectiveness of NPIs [17-19], and that cultural dimensions should be considered when implementing NPIs [17]. Although these studies identify the importance of cultural dimensions in controlling the COVID-19 pandemic, to our knowledge, this work is the first to complement cultural dimensions with NPIs to forecast future COVID-19 infection growth. We recognize that various cultural dimension models exist, such as the six Hofstede cultural dimensions [58], Global Leadership and Organizational Effectiveness (GLOBE) [59], and the Cultural Value Scale (CVSCALE) [60], and that each model has their advocates and criticisms [61]. In this work, we selected the 2015 edition of the Hofstede model [62] due to the relevance of its cultural dimensions in the mentioned studies [17-19,55-57].

Machine learning has been used in applications to combat the COVID-19 pandemic, such as in patient monitoring and genome sequencing [63-66]. Recent studies have also used various statistical and machine learning techniques for short-term forecasting of infection rates for the COVID-19 pandemic [13,15,16,30,33] using reported transmission and mortality statistics, population geographical movement data, and media activity. Pinter et al [13] combined multilayer perceptron with fuzzy inference to predict reported infection and mortality numbers in Hungary with only case number features from May to August 2020. Although reported infection and mortality case numbers aligned with their predictions for May 2020, comparison of the predictions with actual reported numbers from June to August 2020 suggest inaccuracies. Liu et al [15] used internet and news activity predictors within a clustering machine learning model for reported COVID-19 case numbers within Chinese provinces. However, the predictors used within this work are heavily limited to Chinese populations (eg, Baidu search and mobility data, Chinese media sources), and they only predicted cases 2 days ahead. Malki et al [14] used weather, temperature, and humidity features as predictors for COVID-19 mortality rates in regressor machine learning models. Their results suggest that these predictors are relevant for COVID-19 mortality rate modelling. Similar to our work, Saba et al [16] implemented multiple machine learning models to forecast COVID-19 cases based on NPI implementation. However, their work differs in that it only includes lockdown type as an NPI feature (and does not consider cultural dimensions), the study is limited to 9 countries, and the reported case numbers are predicted instead of the change in case numbers. To our knowledge, no other studies have combined NPI and cultural dimension features to predict the growth of reported COVID-19 cases using machine learning. Furthermore, only this work forecasts COVID-19 growth as a measure of CIG (ie, 14-day growth in the cumulative number of reported cases at a national level) across 114 countries via three validation methods, each of which is applicable to a different use case of the model.

### Description of the Study

Due to its direct inference from the number of reported cases, the CIG is a verifiable metric, and it may have a greater impact on the public perception of the magnitude of the COVID-19 pandemic than the actual transmission rate. In this work, CIG reflects the growth in the total number of reported cases within a country in 14 days relative to the total number of previously reported infections, including recoveries and mortalities. We selected 14 days as a suitable period for measuring the change in reported cases because of the expected incubation period of COVID-19. Researchers have found that 97.5% of reported patients with identifiable symptoms developed symptoms within 11.5 days, and 99% developed symptoms within 14 days [67]. We therefore propose the use of 14 days, or 2 weeks, as a suitable period to observe changes in reported case numbers occurring after the implementation of NPIs. A shorter period may lead to the misleading inclusion of reported infections that occurred prior to the implementation of an NPI. Results for a longer period may be misleading as well, given the higher likelihood of change in NPIs within this period that will not be accounted for during prediction. We propose that the CIG over 14 days is a suitable metric that enables inference of the effect of NPIs while being within a relevant period for short-term epidemiology forecasting. We emphasize that the reported number of infections may not necessarily be correlated with the actual transmission rate due to factors such as different testing criteria and varying accessibility in testing over time.

We deployed five machine learning models to predict the CIG for individual countries across 14 days. Explicitly, this value was the label (ie, dependent variable) we sought to predict. We used features (ie, independent variables) representing the implementation levels of NPIs and the cultural dimensions of each country. We obtained daily metrics for the implementation of NPIs at the national level from the OxCGRT data set [1]. Although different countries may implement similar NPIs, researchers have suggested that cross-cultural variations across populations lead to different perceptions and responses toward these NPIs [53,54,68]. We intended to capture any effects due to national cross-cultural differences by complementing the OxCGRT data set with national cultural norm values from the Hofstede cultural dimensions [58]. Our non–time series deep learning models predicted the expected future national CIG using both NPI implementation and cultural norm features. Although time series deep learning models (eg, recurrent neural networks or transformers) may also provide CIG predictions, these models generally require greater amounts of accurately labeled trajectory data and assume that past trajectory trends are readily available representatives of future trajectories. Instead, our non–time series models were trained on more granular data that did not necessarily need to be temporally concatenated into a trajectory. We also opted for less complex non–time series models due to indeterminacies in acquiring and verifying sufficient trajectory data, especially due to the lack of reliable data at the onset of the COVID-19 outbreak.

Our results suggest that non–time series machine learning models can predict future CIG according to multiple validation methods, depending on the user's application. Although we do not necessarily claim state-of-the-art performance for infection rate prediction given the rapidly growing amount of parallel work in this area, to the best of our knowledge, our work is the first to use machine learning techniques to predict the change in national cumulative numbers of reported COVID-19 infections by combining NPI implementation features with national cultural features.

Our implementation uses publicly available data retrieved from the internet and relies on the open-sourced Python libraries Pandas [69] and Scikit-Learn [70].

## Methods

### Data and Preprocessing

Candidate features at the national level were extracted from three data sets for input into our machine learning models: NPIs, cultural dimensions, and current confirmed COVID-19 case numbers.

OxCGRT provides daily level metrics of the NPIs implemented by countries [1]. This data set sorts NPIs into 17 categories, each with either an ordinal policy level metric ranging from 0 (not implemented) to 2, 3, or 4 (strictly enforced) or a continuous metric representing a monetary amount (eg, research funding). The value of each national NPI metric is assigned daily from data in publicly available sources by a team of Oxford University staff and students using the systematic format described in [1]. We limited our candidate features to the 13 ordinal policy categories and 4 computed indices, which represent the implementation of different policy types taken by governments, based on the implemented NPIs. This data set contains data starting from January 1, 2020.

To represent cultural differences across populations of different countries, the 2015 edition of the Hofstede cultural dimensions [62,71] was tagged to each country. Although these dimensions are rarely used in epidemiology studies, they have been used frequently in international marketing studies and cross-cultural research as indicators of the cultural values of national populations [61,72]. Multiple studies have also linked cultural dimensions to health care–related behavior, such as antibiotic usage and body mass index [73-76]. Because the 2015 edition of this data set groups certain geographically neighboring countries together (eg, Ivory Coast, Burkina Faso, Ghana, etc, into Africa West), we tagged all subgroup countries with the dimension values of their group. Although we recognize that this approach is far from ideal and will likely lead to some degree of inaccurate approximation in these subgroup countries, we performed this preprocessing step to include those countries in our study. The dimension values for each country were constant across all samples. Six cultural dimensions were presented for each country or region [71]:

- Power distance index: the establishment of hierarchies in society and organizations and the extent to which lower hierarchical members accept inequality in power
- Individualism versus collectivism: the degree to which individuals are not integrated into societal groups, such as individual or immediate family (individualistic) versus extended families (collectivistic)
- Uncertainty avoidance: a society's tendency to avoid uncertainty and ambiguity through use of societal disapproval, behavioral rules, laws, etc
- Masculinity versus femininity: Societal preference toward assertiveness, competitiveness, and division in gender roles (masculinity) compared to caring, sympathy, and similarity in gender roles (femininity)
- Long-term versus short-term orientation: Societal values toward tradition, stability, and steadfastness (short-term) versus adaptability, perseverance, and pragmatism (long-term)
- Indulgence versus restraint: The degree of freedom available to individuals for fulfilling personal desires by social norms, such as free gratification (indulgence) versus controlled gratification (restraint)

We extracted the daily number of confirmed cases, *n_t_*, for each country from the COVID-19 Data Repository by the CSSE at Johns Hopkins University [34]. We used a rolling average of the previous 5-day window to smooth fluctuations in *n_t_*, which may be caused by various factors, such as inaccurate case reporting, no release of confirmed case numbers (eg, on weekends and holidays), and sudden infection outbreaks. We refer to the smoothed daily number of confirmed cases for date *t* as 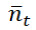.

We computed the CIG for a specified date, *τ*, as:


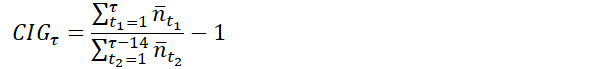


The CIG represents the expected number of new confirmed cases from date *τ* – 13 to date *τ* as a percentage of the total number of confirmed infection cases up to date *τ* – 14.

Our goal was to predict the CIG 14 days in advance (ie, *CIG*_τ+14_) given information from the current date *τ* for each country. Available candidate features included all ordinal policy metrics and the four computed indices from OxCGRT, the six cultural dimension values from the Hofstede model, the CIG of the current date *CIG_τ_*, and the smoothed cumulative number of confirmed cases 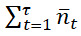, for a total of 25 candidate features. Neither the date nor any other temporal features were included.

We trimmed samples with fewer than 10 cumulative confirmed infection cases and with the highest 2.5% and the lowest 2.5% of *CIG*_τ+14_ to remove outliers in the data. Because the lowest 2.5% of *CIG*_τ+14_ were all 0.0%, we removed the samples with *CIG*_τ+14_=0.0% by ascending date.

Our data range from April 1 to September 30, 2020, inclusively. We excluded all countries from our combined data set that had missing feature values. In total, our combined data set and our experiments applied to 114 countries: Algeria, Angola, Argentina, Australia, Austria, Bahrain, Bangladesh, Belgium, Benin, Botswana, Brazil, Bulgaria, Burkina Faso, Burundi, Cameroon, Canada, Central African Republic, Chad, Chile, China, Colombia, Comoros, Croatia, Czech Republic, Denmark, Djibouti, Egypt, El Salvador, Eritrea, Estonia, Ethiopia, Finland, France, Gabon, Gambia, Germany, Ghana, Greece, Guinea, Hong Kong, Hungary, India, Indonesia, Iran, Iraq, Ireland, Italy, Japan, Jordan, Kenya, Kuwait, Latvia, Lebanon, Lesotho, Liberia, Libya, Lithuania, Luxembourg, Madagascar, Malawi, Malaysia, Mali, Mauritania, Mauritius, Mexico, Morocco, Mozambique, Namibia, Netherlands, New Zealand, Niger, Nigeria, Norway, Oman, Pakistan, Palestine, Peru, Philippines, Poland, Portugal, Qatar, Romania, Russia, Rwanda, Saudi Arabia, Senegal, Serbia, Seychelles, Sierra Leone, Singapore, Slovenia, Somalia, South Sudan, Spain, Sudan, Sweden, Switzerland, Syria, Taiwan, Tanzania, Thailand, Togo, Trinidad and Tobago, Tunisia, Turkey, Uganda, United Arab Emirates, United States, Uruguay, Venezuela, Vietnam, Yemen, Zambia, and Zimbabwe.

The mean, standard deviation, and range of each candidate feature value for the above countries are shown in Table 1.

**Figure 1:**
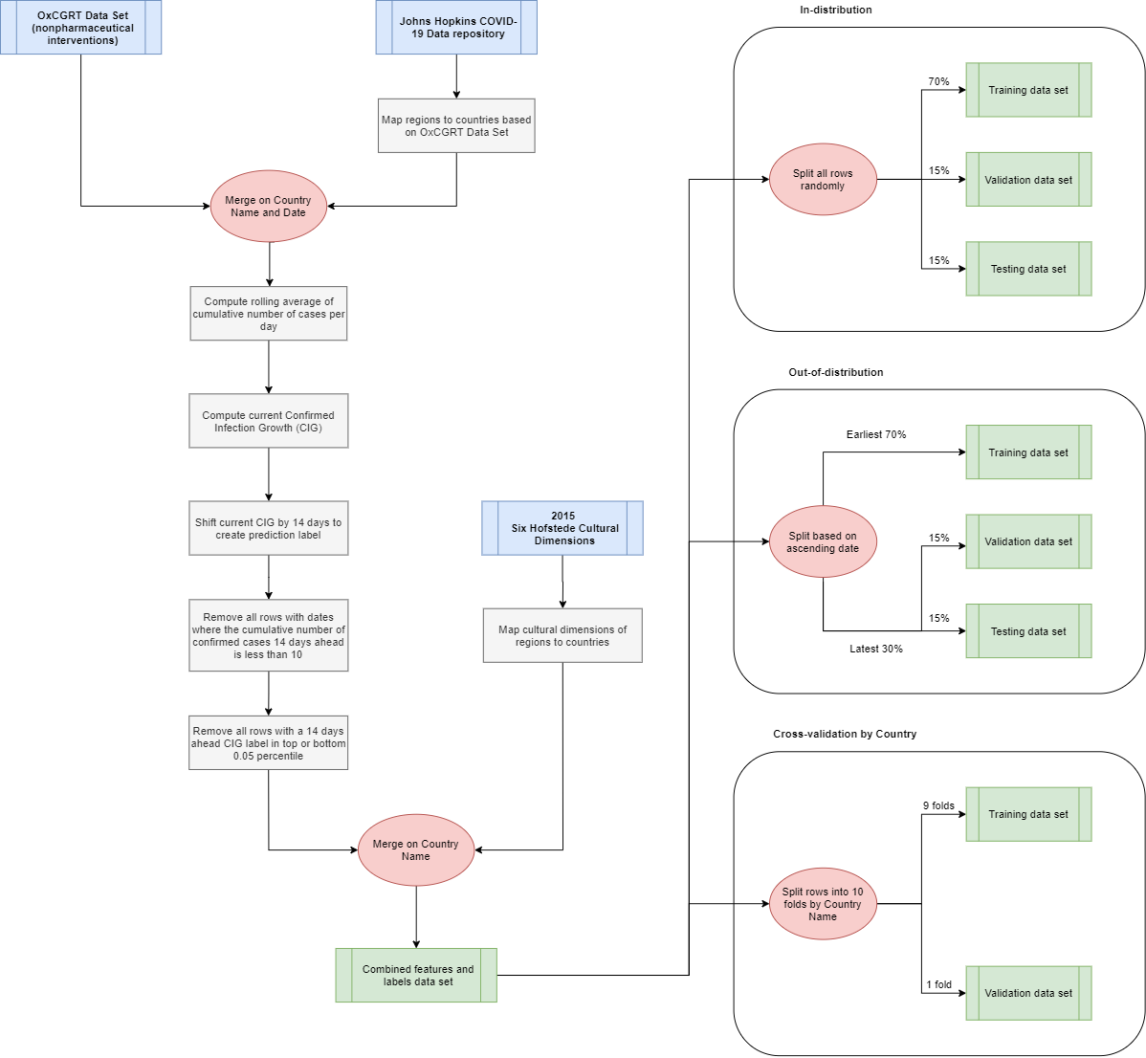
Data preprocessing pipeline from the OxCGRT data set, Johns Hopkins COVID-19 Data Repository, and six Hofstede cultural dimensions to the training, validation, and test data sets for each validation method. OxCGRT: Oxford COVID-19 Government Response Tracker.

The data preprocessing procedure is shown in Figure 1.

**Table 1:** Statistical measurements of candidate feature values.

Candidate features		Mean (SD)	Range
**Nonpharmaceutical interventions**			
	School closure	2.23 (1.01)	0.00 to 3.00
	Workplace closure	1.67 (0.92)	0.00 to 3.00
	Cancellation of public events	1.64 (0.65	0.00 to 2.00
	Restrictions on gatherings	2.89 (1.27)	0.00 to 4.00
	Closure of public transport	0.71 (0.77)	0.00 to 2.00
	Stay-at-home requirements	1.17 (0.90)	0.00 to 2.00
	Restrictions on internal movement	1.15 (0.88)	0.00 to 2.00
	International travel controls	3.13 (1.00)	0.00 to 4.00
	Income support	1.04 (0.79)	0.00 to 2.00
	Debt/contract relief	1.23 (0.76)	0.00 to 2.00
	Public information campaigns	1.97 (0.23)	0.00 to 2.00
	Testing policy	1.84 (0.82)	0.00 to 2.00
	Contact tracing	1.50 (0.64)	0.00 to 2.00
	Stringency Index	63.02 (20.57)	0.00 to 100.00
	Government Response Index	61.43 (15.03)	0.00 to 95.54
	Containment Health Index	62.91 (16.50)	0.00 to 98.96
	Economic Support Index	52.53 (28.93)	0.00 to 100.00
**Current infection numbers**			
	Current cumulative number of confirmed cases: 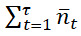	113,302.24 (505,170.50)	4.00 to 7,155,220.00
	*CIG*_τ_^a^	0.85 (3.83)	–0.423 to 228.00
**Hofstede cultural dimensions**			
	Power distance	66.74 (17.34)	11.00 to 104.00
	Individualism	38.52 (18.71)	12.00 to 91.00
	Masculinity	48.32 (14.06)	5.00 to 95.00
	Uncertainty avoidance	64.17 (17.42)	8.00 to 112.00
	Long-term orientation	35.36 (21.52)	3.52 to 92.95
	Indulgence	46.88 (20.47)	0.00 to 100.00

^a^*CIG*_τ_: confirmed infection growth on the current day.

### Feature Selection and Processing

We selected features to input into our machine learning models from our candidate feature pool using mutual information [77]. Mutual information is a measure of the dependency between an individual feature (ie, the independent variable) and the label (ie, the dependent variable), and it captures both linear and nonlinear dependencies. However, mutual information does not capture multivariate dependencies or indicate collinearity between features. To include both linear and nonlinear dependencies, features are selected if they achieve substantially nonzero mutual information (ie, greater than 0.10). Feature selection was conducted prior to training with the training set in all validation methods. Similar feature filtering and selection techniques have been used in other machine learning applications [70,78]. The candidate features considered for input and their respective mutual information are listed in Table 2 for the in-distribution and out-of-distribution validation methods. Mutual information was also computed for each of the ten folds of the cross-validation method.

All selected features were then normalized to the range [0,1] using standard min-max normalization.

**Table 2:** Mutual information of candidate features for the in-distribution and out-of-distribution validation methods. In the cross-validation method, the 10 folds have varying mutual information.

Candidate feature			Mutual information		
			In-distribution		Out-of-distribution
**Nonpharmaceutical interventions**					
	School closure^a,b^	0.184		0.205	
	Workplace closure^b^	0.098		0.127	
	Cancellation of public events^b^	0.089		0.127	
	Restrictions on gatherings^a,b^	0.107		0.112	
	Closure of public transport^b^	0.094		0.124	
	Stay-at-home requirements^a,n^	0.139		0.163	
	Restrictions on internal movement^a,b^	0.126		0.146	
	International travel controls	0.099		0.099	
	Income support^b^	0.095		0.110	
	Debt/contract relief	0.043		0.053	
	Public information campaigns	0.020		0.023	
	Testing policy	0.056		0.064	
	Contact tracing	0.030		0.038	
	Stringency Index^a,b^	0.638		0.668	
	Government Response Index^a,b^	0.634		0.641	
	Containment Health Index^a,b^	0.621		0.655	
	Economic Support Index^a,b^	0.119		0.124	
**Current infection numbers**					
	Current cumulative number of confirmed cases: 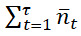 ^a,b^	0.517		0.557	
	*CIG*_τ_^a,b,c^	0.866		0.798	
**Hofstede cultural dimensions**					
	Power distance^a,b^	0.288		0.342	
	Individualism^a,b^	0.309		0.355	
	Masculinity^a,b^	0.310		0.372	
	Uncertainty avoidance^a,b^	0.314		0.370	
	Long-term orientation^a,b^	0.461		0.535	
	Indulgence^a,b^	0.456		0.529	

^a^Selected feature for the in-distribution method.

^b^Selected feature for the out-of-distribution method.

^c^*CIG*_τ_: confirmed infection growth on the current day.

### Model Training and Validation

We trained the machine learning models by performing a grid search over the combinations of hyperparameters listed in Table 3 [70,79-82]. We optimized the models using the mean squared error (MSE) criterion and selected the model hyperparameters with the lowest mean absolute error (MAE) as the optimal configuration of the model. The MSE heavily penalizes large residual errors disproportionately, while the MAE provides an absolute mean of all residual errors [83]. The MAE of the training data acts as a measure of the goodness-of-fit of the model, while the MAE of the validation and testing data acts as a measure of the predictive performance [84].

**Table 3:** Machine learning models and hyperparameter combinations used in the grid search.

Model		Hyperparameters
**Ridge regression**		
	α	0.00, 0.25, 0.50, 0.75, 1.00, 1.25
**Decision tree regression**		
	Depth	5, 10, 15, 20, 25, 30
	Minimum sample split	2, 5, 10
	Minimum sample leaves	1, 2, 4, 8, 10
**Random forest regression**		
	Depth	5, 10, 20, 25, 30
	Estimators	3, 5, 10, 15, 20, 30, 50, 75, 100, 125, 150
	Minimum sample split	2, 5, 10
	Minimum sample leaves	1, 2, 4, 8, 10
**AdaBoost^a^ regression**		
	Weak learner	Decision tree (maximum depth: 2)
	Estimators	3, 5, 10, 15, 20, 30, 50, 75, 100, 125, 150
	Loss function	Linear
	Learning rate	0.1, 0.5, 1.0
**Support vector regression**		
	ε	0.00, 0.10, 0.20, 0.50
	Kernel	Linear, radial, sigmoid

^a^AdaBoost: adaptive boosting.

To validate in-distribution and out-of-distribution, we split our samples into 70-15-15 training-validation-test sets. For cross-validation [85,86], we split our samples into 10 folds (ie, 90-10). These three methods of validation each represent a different definition of performance for the machine learning models.

#### In-Distribution Validation

We randomly split the samples into training, validation, and test sets. Consequently, the models were trained from samples distributed across the entire date range available in our data. This is critical, as it is generally expected that model performance is best when training and test data are drawn from the same distribution. Because the COVID-19 infection numbers naturally constitute a time series, this method ensures that validation and test samples are indeed from the same distribution as the training samples. Because the samples are disassociated from their dates and all other known temporal features, the prediction of the validation and test samples using the training samples is unordered. This method may be applicable to use cases in which the date-to-predict is expected to be in a similar distribution as the training samples, such as predicting *CIG*_τ+14_ when data up to the current date *τ* are available.

#### Out-of-Distribution Validation

Although the in-distribution method can ensure that the training, validation, and test data are all sampled from the same distribution, it may not necessarily be the most practical method. Generally, the goal of long-term infection rate forecasting is to anticipate future infection rates, and it should not be represented as an in-distribution task, where we trained it with data from near or later than the date-to-predict. Therefore, we also validated the performance of our models by training on the earliest 70% of the samples. The validation and test sets were then randomly split between the remaining 30% of the samples. This setup ensures that all training samples occurred earlier than the validation and testing samples and that no temporal features (known or hidden) were leaked. However, due to the changing environment related to COVID-19 infections (eg, the introduction of new NPIs, seasonal changes, new research), the validation and testing distributions are likely different from that of the training set. This method may be applicable for use cases in which the date-to-predict is in the far future and not all data up to 14 days prior to the date-to-predict are available.

#### Country-Based Cross-Validation

As a compromise between the above two methods, we also used a cross-validation method in which we split the available countries into 10 folds. The aim was to evaluate validation samples from the same date range as the training samples, but not the same country trajectory. That is, only data from countries not in the validation set are included in the training set. Although the samples from the training and validation sets are therefore sampled from different distributions (ie, different countries), we anticipate that features from the Hofstede cultural dimensions [58] may assist in identifying similar characteristics between countries, thus reducing the disparity between the training and validation distributions. This method may be applicable in predicting the CIG of countries for which previous associated data is unavailable or unreliable.

## Results

### Feature Selection

For both the in-distribution and out-of-distribution training sets, we observed that most candidate features met our requirement of nonzero mutual information (≥0.10) (see Table 2).

In both training sets, the candidate features that did not meet the requirements were international travel control (0.099, 0.099), debt/contract relief (0.043, 0.053), public information campaigns (0.020, 0.023), testing policy (0.056, 0.064), and contact tracing (0.030, 0.038). Additional candidate features that did not meet the requirements for the in-distribution training set were workplace closure (0.098) and cancellation of public events (0.089). Overall, the in-distribution and out-of-distribution data sets contained 17 and 20 features, respectively.

*CIG*_τ_ had the highest mutual information out of all features, suggesting similarities between the feature *CIG*_τ_ and the label *CIG*_τ+14_. Further analysis showed a correlation of *r*=.309 between *CIG*_τ_ and *CIG*_τ+14_. This may be due to similar trends in the CIG when the implementation of NPIs is consistent within a 14-day period. We also observed that all candidate features for the six Hofstede cultural dimensions had higher mutual information than all individual NPI candidate features, aside from the aggregated indices. This finding suggests a high statistical relationship between each cultural dimension feature and the label we sought to predict. Although the cultural dimension values may not fully represent the cultural differences of each country (see Limitations), there is sufficient information between each cultural dimension feature and the label for them to be relevant predictors of the label.

### Comparison of Machine Learning Models

Out of all the available configurations (ie, hyperparameter combinations) of each model, we selected the model configurations with the lowest validation errors and computed the test errors. The parameters for these selected models are listed in Table 4. The mean training, validation, and test errors are included in Table 5, Table 6, and Table 7, respectively, for the in-distribution, out-of-distribution, and cross-validation methods. We also include the median percent error [87], which is the percentage difference of the prediction *f*(*x*^(^*^i^*^)^) and the label *y*^(^*^i^*^)^ for each instance {*x*^(^*^i^*^)^_,_ *y*^(^*^i^*^)^}, computed as:


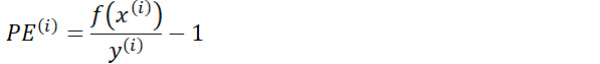


We observed that random forest regression had the lowest mean test error in the interpolation method (0.031) and adaptive boosting (AdaBoost) regression had the lowest mean test errors in the extrapolation and cross-validation methods (0.089 and 0.167, respectively) (see Table 5, Table 6, and Table 7). For all models aside from ridge regression, the in-distribution method had the lowest mean test errors and the lowest median percent error.

**Table 4:** Hyperparameters of the optimal configuration (lowest validation mean absolute error) for each model for each validation method.

Model		Validation method		
		In-distribution	Out-of-distribution	Cross-validation
**Ridge regression**				
	α	0.00	0.25	0.00
**Decision tree regression**				
	Depth	25	10	5
	Minimum sample split	2	5	2
	Minimum sample leaves	1	1	4
**Random forest regression**				
	Depth	30	15	15
	Estimators	150	10	125
	Minimum sample split	2	2	2
	Minimum sample leaves	1	10	10
**AdaBoost^a^ regression**				
	Estimators	5	5	3
	Learning rate	0.1	1.0	0.1
**Support vector regression**				
	ε	0.00	0.00	0.00
	Kernel	Radial	Linear	Linear

^a^AdaBoost: adaptive boosting.

**Table 5:** Optimal MAE and median percent error values for the in-distribution validation method.

Model	Train MAE^a^	Validation MAE	Test MAE	Validation percent error	Test percent error
Ridge regression	0.270	0.269	0.259	1.58	0.60
Decision tree regression	0.001	0.041	0.039	1.00	0.00
Random forest regression^b^	0.012	0.033	0.031	1.01	1.01
AdaBoost^c^ regression	0.162	0.166	0.155	1.31	1.24
Support vector regression	0.170	0.172	0.165	1.00	1.01

^a^MAE: mean absolute error.

^b^The model with the lowest test MAE.

^c^AdaBoost: adaptive boosting.

**Table 6:** Optimal MAE and median percent error values for the out-of-distribution validation method.

	Train MAE^a^	Validation MAE	Test MAE	Validation percent error	Test percent error
Ridge regression	0.296	0.240	0.247	2.26	1.22
Decision tree regression	0.117	0.109	0.114	1.15	0.12
Random forest regression	0.098	0.098	0.105	1.45	0.44
AdaBoost^b^ regression^c^	0.207	0.081	0.089	1.40	0.39
Support vector regression	0.268	0.167	0.176	1.66	0.60

^a^MAE: mean absolute error.

^b^AdaBoost: adaptive boosting.

^c^The model with the lowest test MAE.

**Table 7:** Optimal MAE and median percent error values for the cross-validation method. Validation error is equivalent to test error for cross-validation.

Model	Train MAE^a^	Validation MAE	Validation percent error
Ridge regression	0.262	0.275	0.62
Decision tree regression	0.181	0.207	0.28
Random forest regression	0.073	0.175	0.40
AdaBoost^b^ regression^c^	0.164	0.167	0.27
Support vector regression	0.230	0.240	0.03

^a^MAE: mean absolute error.

^b^AdaBoost: adaptive boosting.

^c^The model with the lowest test MAE.

### Analysis of Best-Performing Models

**Figure 2:**
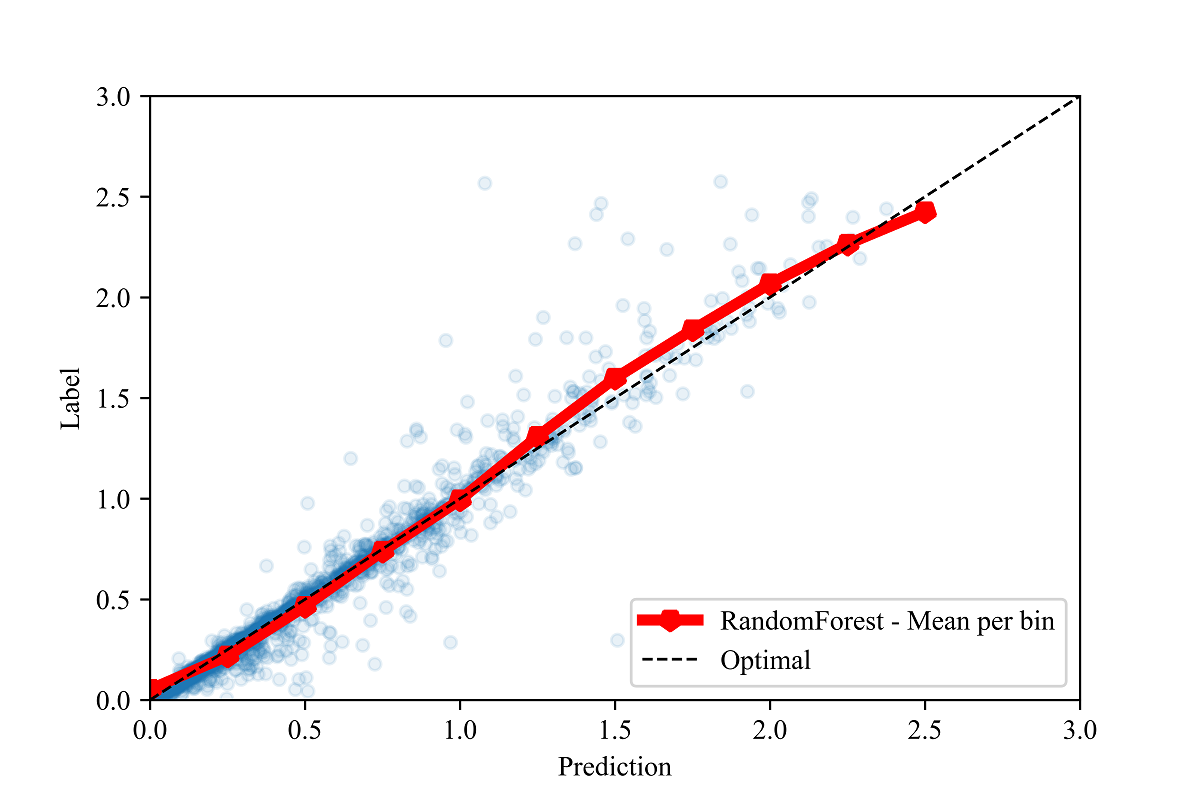
Calibration plot between the labels and predictions for the interpolation validation method, with the mean of each prediction bin of size 0.25.

**Figure 3:**
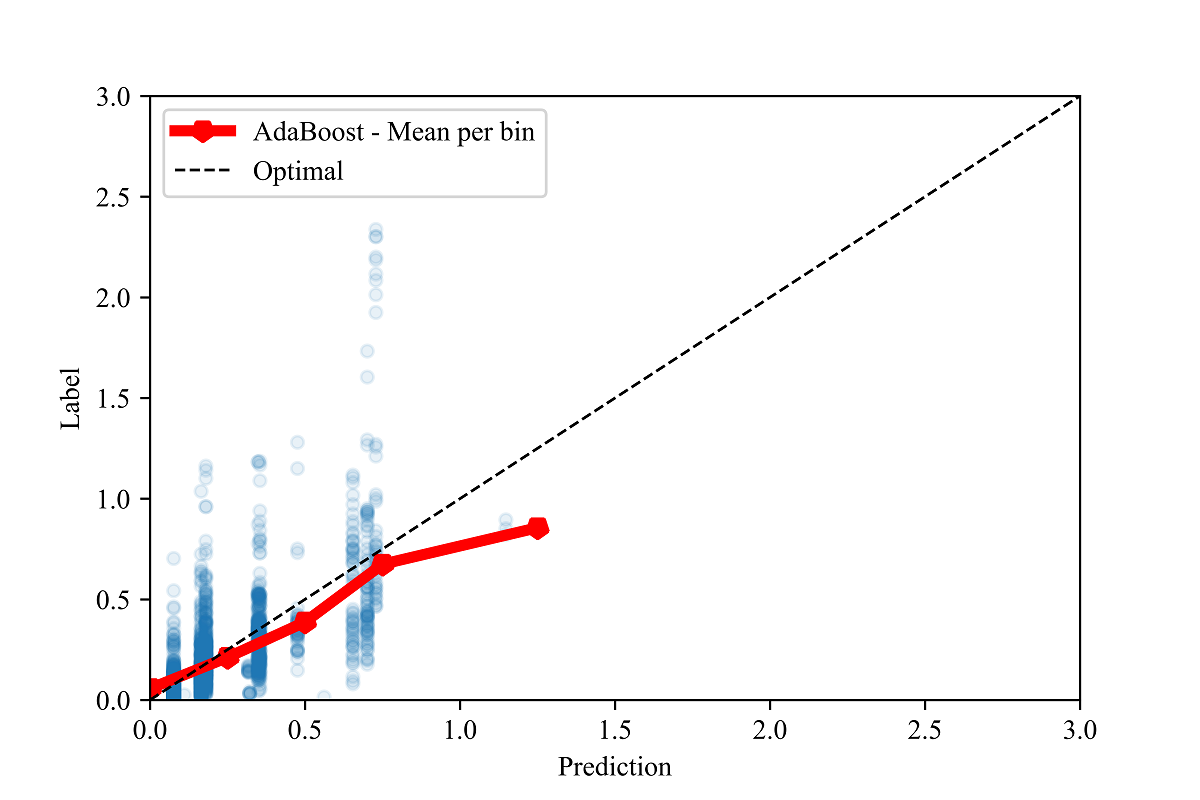
Calibration plot between the labels and predictions for the extrapolation validation method, with the mean of each prediction bin of size 0.25.

**Figure 4:**
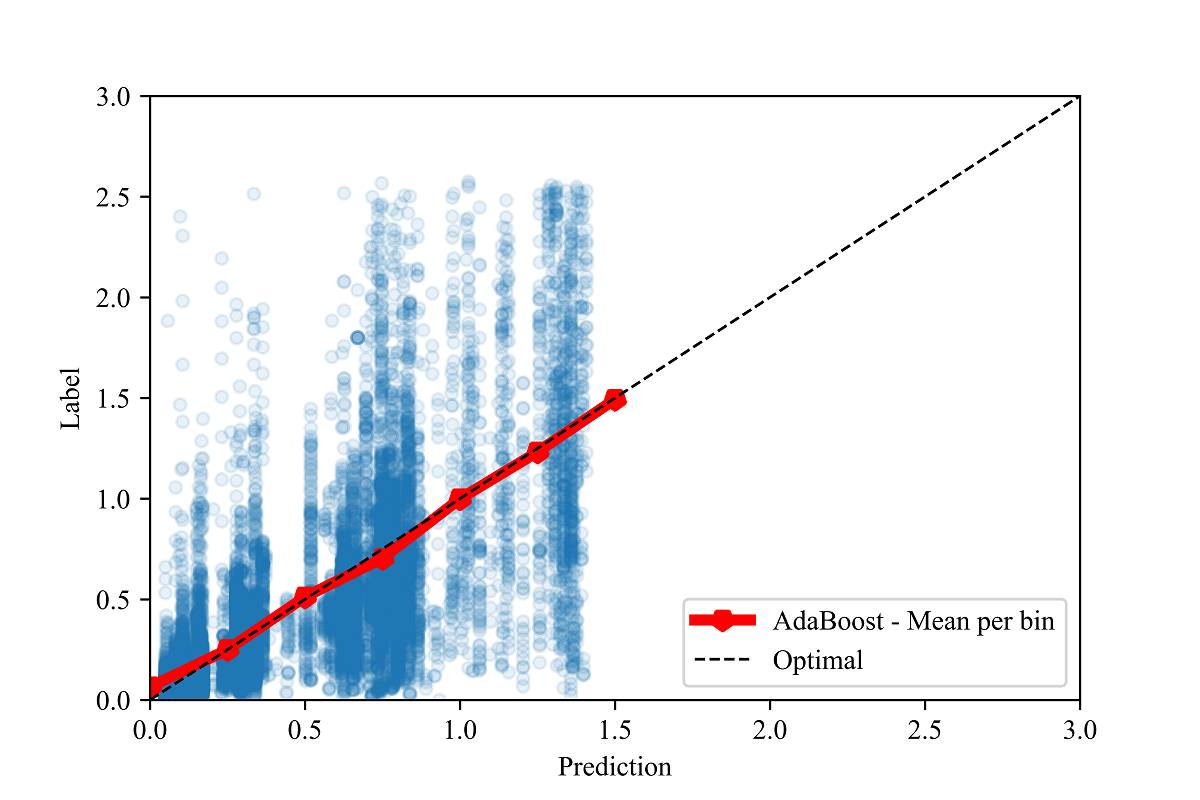
Calibration plot between the labels and predictions for the cross-validation method, with the mean of each prediction bin of size 0.25.

Intercepts near 0.0 and slopes near 1.0 are the linear calibration measures that indicate a perfect calibration relationship between the predictions and the labels [84]. For the optimal models in all the validation methods, we observed slopes close to 1.0 and intercepts close to 0.0 (see Table 8). Due to the large sample sizes, statistical significance testing indicated that several slopes and intercepts are significantly different from 1.0 and 0.0, respectively. However, the small mean differences (standardized to the standard deviation, ie, the z score) indicate that these differences have no practical significance. High correlations ( *r*>0.70) and moderate-to-high *R*^2^ values (*R*^2^>.50) [88,89] between the predictions and labels were observed in all three validation methods (see Figure 2, Figure 3, and Figure 4).

**Figure 5:**
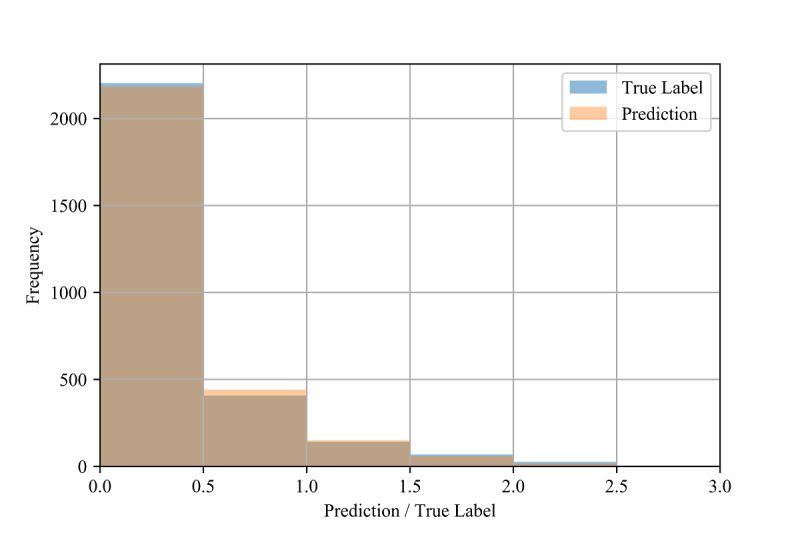
Distributions of the test labels (ie, true confirmed infection growth) and model predictions (n=2847) for the in-distribution method.

**Figure 6:**
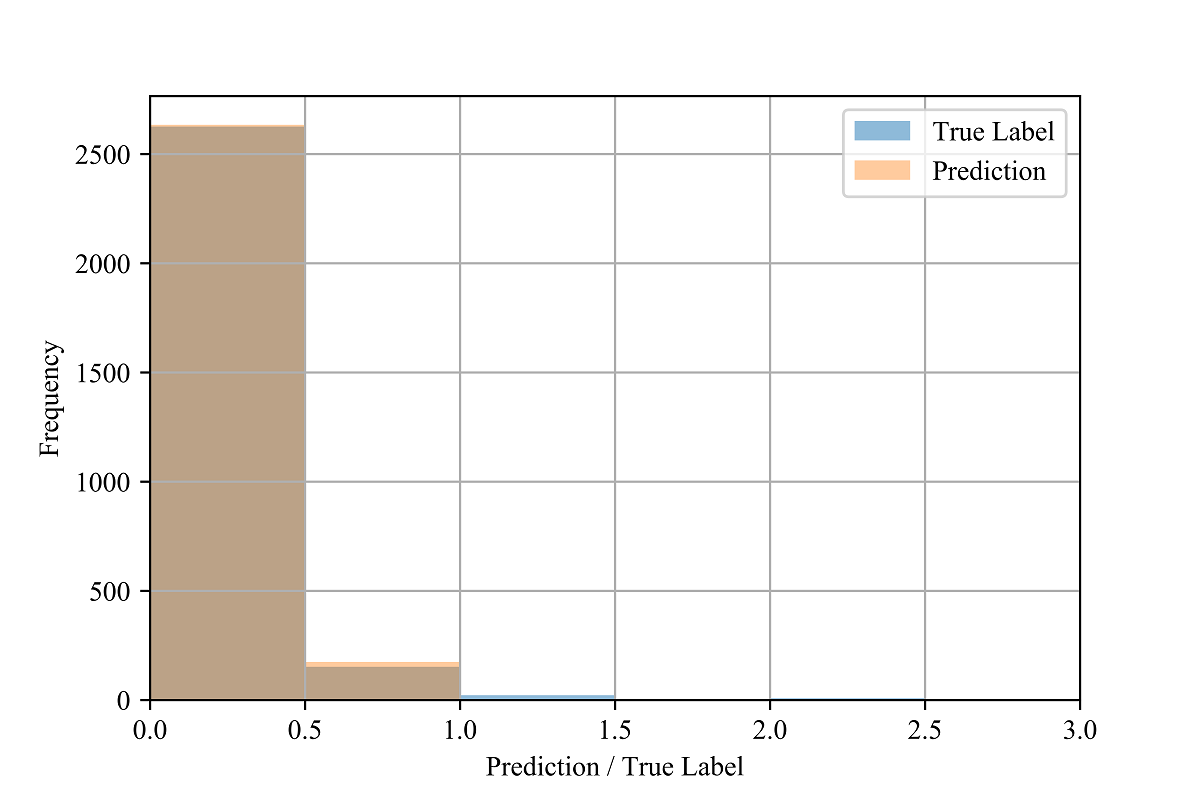
Distributions of the test labels (ie, true confirmed infection growth) and model predictions (n=2811) for the out-of-distribution method.

**Figure 7:**
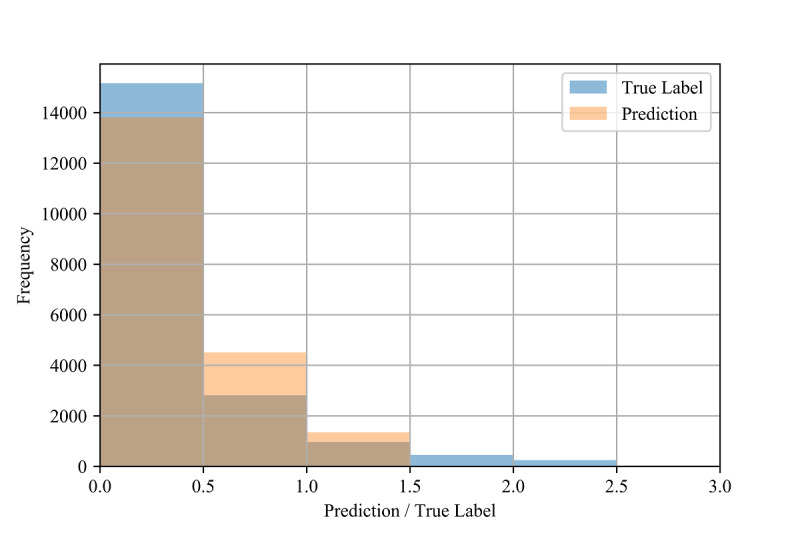
Distributions of the test labels (ie, true confirmed infection growth) and model predictions (n=19,669) for the cross-validation method.

To assess the fine-grained model performance, we discretized both the true labels and model predictions into bins of size 0.5 for all three validation methods (see Figure 5, Figure 6, and Figure 7). Comparing the resulting empirical distributions, it can be seen that the resulting distributions are extremely similar in both the in-distribution and out-of-distribution methods. In the cross-validation method, the predictions skew slightly higher than the labels in the 0.0-1.0 range, showing a general tendency of the model to slightly overestimate the CIG within this range.

Further analysis shows that the performance of the models varies with the values of the labels. In both the in-distribution and cross-validation methods, the test MAE is lowest for samples with labels of 0.0 (see Table 9 and Table 10), followed by the label range of 0.0-0.5. In the out-of-distribution method, the test MAE is lowest for samples with labels from 0.0-0.5 (see Table 11). For all validation methods, the mean MAE and median percent errors also increase with label bins greater than 1.0, showing a decrease in accuracy for a larger CIG.

**Table 8:** Linear calibration measures of the models with the lowest test mean absolute error for each validation method.

Measure	Validation method		
	In-distribution	Out-of-distribution	Cross-validation
Test sample size, n	2847	2811	19,669
Model	Random forest	AdaBoost^a^	AdaBoost
Correlation, *r*	0.979	0.716	0.758
Slope (SE)	1.037 (0.004)	0.986 (0.018)	0.968 (0.006)
Slope standardized mean difference (z score) from 1	0.176	–0.015	–0.039
Slope *P* value (mean of 1)	<.001	.43	<.001
Intercept (SE)	–0.013 (0.002)	–0.011 (0.004)	0.006 (0.003)
Intercept standardized mean difference (z score) from 0	–0.119	–0.044	0.014
Intercept *P* value (mean of 0)	<.001	.02	.06
*R*^2^ value	0.959	0.513	0.574

^a^AdaBoost: adaptive boosting.

**Table 9:** Test errors and median percent errors of label bins of size 0.5 for the in-distribution validation method.

Upper threshold	Count	Test mean nonabsolute error (SD)	Test mean absolute error (SD)	Test percent error
0.0	20	0.000 (0.000)	0.000 (0.000)	N/A^a^
0.5	2183	0.011 (0.052)	0.017 (0.050)	0.01
1.0	408	0.003 (0.076)	0.047 (0.060)	0.00
1.5	140	–0.052 (0.139)	0.094 (0.115)	–0.02
2.0	68	–0.104 (0.205)	0.158 (0.167)	–0.04
2.5	26	–0.283 (0.309)	0.297 (0.294)	–0.08
3.0	2	–1.108 (0.470)	1.108 (0.470)	–0.43

^a^N/A: not applicable.

**Table 10:** Test errors and median percent errors of label bins of size 0.5 for the cross-validation method.

Upper threshold	Count	Test mean nonabsolute error (SD)	Test mean absolute error (SD)	Test percent error
0.0	114	–0.059 (0.086)	0.059 (0.086)	N/A^a^
0.5	15,056	–0.073 (0.174)	0.109 (0.153)	0.493
1.0	2815	–0.010 (0.282)	0.217 (0.181)	–0.006
1.5	960	0.333 (0.337)	0.393 (0.265)	–0.299
2.0	451	0.719 (0.370)	0.719 (0.370)	–0.391
2.5	246	1.141 (0.321)	1.141 (0.321)	–0.459
3.0	27	1.362 (0.266)	1.225 (0.266)	–0.486

^a^N/A: not applicable.

**Table 11:** Test errors and median percent errors of label bins of size 0.5 for the out-of-distribution validation method.

Upper threshold	Count	Test mean nonabsolute error (SD)	Test mean absolute error (SD)	Test percent error
0.0	19	0.076 (0.000)	0.076 (0.000)	N/A^a^
0.5	2607	0.034 (0.096)	0.071 (0.074)	0.44
1.0	152	–0.161 (0.228)	0.225 (0.164)	–0.25
1.5	22	–0.648 (0.222)	0.648 (0.222)	–0.52
2.0	3	–1.044 (0.147)	1.044 (0.147)	–0.60
2.5	8	–1.464 (0.116)	1.464 (0.116)	–0.67

^a^N/A: not applicable.

## Discussion

### Principal Results

Our results suggest that traditional, non–time series machine learning models can predict future CIG to an appreciable degree of accuracy, as suggested by the moderate-to-high *R*^2^ values (*R*^2^>0.50) and strong linear calibration relationships (*r*>0.70) [88,89] between the labels and predictions in all the validation methods.

A comparison of our results for all the validation methods suggests differences in the predictive performance of the machine learning models across the varying use cases. The in-distribution method has the highest *R*^2^ value and the lowest test mean error and median percent error; this is to be expected, as the test samples were obtained from the same distribution as the training samples. Intuitively, although the samples in the in-distribution method are unordered (ie, no temporal features are included), the availability of samples across the entire temporal range in the training set enables the validation and test samples to interpolate between these training samples.

The out-of-distribution method achieved a higher test mean error and a lower *R*^2^ value than the in-distribution method. This is expected, as the evolving COVID-19 infection trajectories observed in most countries give distributions of training samples from earlier dates that may differ greatly from those of validation and test samples from later dates (ie, data shift), which machine learning models are often ill-equipped to handle [90].

Conversely, although the cross-validation method contained the training and validation sets within the same date range, the cross-validation method also separated countries across these sets (ie, the 10 folds) such that no country had samples in both the training and validation sets. This difference led to higher test mean errors and median percent errors than the other two methods and a similar *R*^2^ value to that of the out-of-distribution method, suggesting that including training samples from the same country as the validation samples is more important than ensuring temporal overlap. We speculate that this result occurs because the unique cultural dimensions per country may potentially act as categorical rather than continuous features for each country. In such cases, the cultural dimensions observed in the training set would be considered irrelevant to the cultural dimensions within the validation set.

The performance also varied depending on the value of the label (see Table 9, Table 10, and Table 11), which may be due to the imbalanced frequency of the training samples. That is, the rareness of samples with higher CIG compared to lower CIG in the training set may be the cause of their comparatively poor performance.

In Figure 3 and Figure 4, we also show constraints of the trained AdaBoost regression models. The discretization of the prediction values may be due to the low number of estimators used in the lowest mean test error configuration, as shown in Table 4. The low number of estimators in these configurations may also restrict the predictions to a maximum of 1.5 selected to the relatively low number of samples with labels greater than 1.5 (see Figure 6 and Figure 7). The label ranges with the most samples are selected over underrepresented ranges as candidates for prediction values in the discretized AdaBoost regression models. Although additional estimators in the AdaBoost regression models may result in less discrete prediction values, they may also cause over-fitting by increasing the complexity of the models.

### Limitations

First, the scores in the OxCGRT and Hofstede cultural dimensions data sets are imprecise. NPI enforcement levels and definitions may vary even between countries with the same scores, while countries sharing similar cultural dimension scores may have unobserved differences in terms of cultural practices due to low representation of their cultures with only six dimensions. Although the Hofstede model is convenient for the goal of our work, it does not identify intracountry cultural differences. Furthermore, distinct countries may be grouped within specific geographical regions (eg, Africa West). We also acknowledge that there are trade-offs between different cultural models and different definitions of culture [61]. We encourage further exploration of appropriate cultural dimensions in addition to the Hofstede model, such as GLOBE [59] and CVSCALE [60]. Second, by predicting the CIG 14 days in advance of the current date, the models do not account for information regarding changes in NPIs between the current date and the date-to-predict. Third, the CIG is a measure of the change in the cumulative number of confirmed infections and may not necessarily be correlated with the change in the daily number of confirmed infections or the actual transmission rate of COVID-19. For example, differences in testing and reporting policies of different jurisdictions (eg, prioritizing high-risk patients, performing more tests per capita, and obfuscating test results) may lead to a misleading representation of the infection growth.

### Conclusion

In this study, we trained five non–time series machine learning models to predict the CIG 14 days into the future using NPI features extracted from the OxCGRT data set [1] and cultural norm features extracted from the Hofstede cultural dimensions [58]. Together, these features enabled the prediction of near-future CIG in multiple machine learning models. Specifically, we observed that random forest regression and AdaBoost regression resulted in the most accurate predictions out of the five evaluated machine learning models.

We observed differences in the predictive performance of the machine learning models across the three validation methods; the highest accuracy was achieved with the in-distribution method and the lowest with the cross-validation method. These differences in performance suggest that the models have varying levels of accuracy depending on the use case. Specifically, predictions are expected to have higher accuracies when existing data from the same country in nearby dates are available (ie, in-distribution method). This enables applications such as predicting the CIG over the upcoming 14 days from the current date. The decrease in accuracy when data from nearby dates are unavailable (ie, the out-of-distribution method) suggests weaker performance in predicting the CIG over 14 days for relatively distanced future dates. We observed the greatest decrease in performance when data from the same country were unavailable (ie, the cross-validation method). However, with all validation methods, we observed appreciable calibration measures between the predictions and labels of the test set.

This study adds to the rapidly growing body of work related to predicting COVID-19 infection rates by introducing an approach that incorporates routinely available data on NPIs and cultural dimensions. Importantly, this study emphasizes the utility of NPIs and cultural dimensions for predicting country-level growth of confirmed infections of COVID-19, which to date have been limited in existing forecasting models. Our findings offer a new direction for the broader inclusion of these types of measures, which are also relevant for other infectious diseases, using non–time series machine learning models. Our experiments also provide insight into validation methods for different applications of the models. As the availability of this data increases and the nature of the data continues to evolve, we expect that models such as these will produce accurate and generalizable results that can be used to guide pandemic planning and other infectious disease control efforts.

## Abbreviations

CIG: confirmed infection growth
CSSE: Center for Systems Science and Engineering
CVSCALE: Cultural Value Scale
GLOBE: Global Leadership and Organizational Effectiveness
MAE: mean absolute error
MSE: mean squared error
NPI: nonpharmaceutical intervention
OxCGRT: Oxford COVID-19 Government Response Tracker
SIR: susceptible-infected-recovered
